# What do you need to know to set up CPD as a professional body?

**Published:** 2017-05-12

**Authors:** Helena P Filipe, Karl Golnik, Daksha Patel

**Affiliations:** 1 Ophthalmology service, Department of surgery, Hospital of the armed forces/PL-EMGFA unit of Ophthalmology, Hospital SAMS Lisbon Street Sergeant José Paulo dos Santos, Lisbon, Portugal.; 2 International Council of Ophthalmology Director for Education. Professor & Chairman, Department of Ophthalmology, University of Cincinnati & the Cincinnati Eye Institute, Ohio, USA.; 3 E-learning Director, International Centre for Eye Health, London School of Hygiene and Tropical Medicine, London, UK.

**Setting up an effective CPD system as a professional body is a complex task that involves advocating the importance to health care professionals, communication with the regulators, setting and maintaining standards and monitoring the process.**

**Figure:**
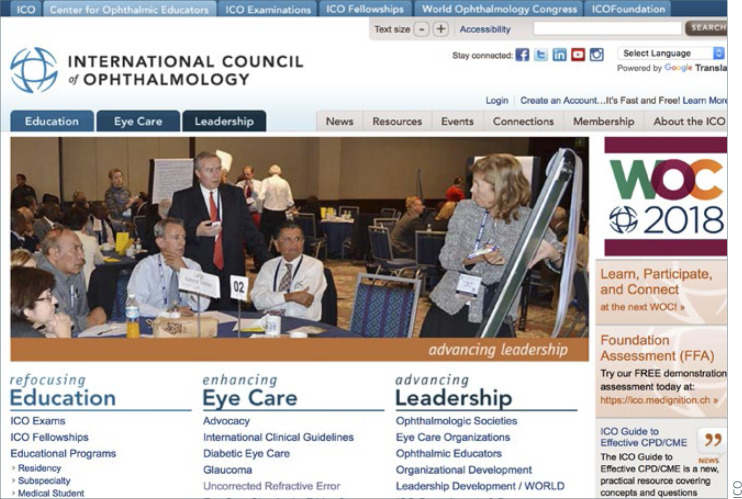
ICO website (screen grab) – an established resource for CPD.

**“We need assessment tools. We need support. We need faculty development. And I think we also need some patience. This is going to take a little bit of time.”**

J. Mark Walton, MD, FRCSC; assistant dean of Postgraduate Medical Education at McMaster University

Continuing Professional Development (CPD) incorporates Continuing Medical Education (CME) as one modality and embraces other relevant wide-ranging educative means and competencies required to practise high quality medicine.

Society has a longstanding social contract with the medical profession. Life-long learning is the scaffold of safe and up-to-date practice which incorporates healthcare workforce accountability. CPD is a cyclic, continuous, self-directed, practice-based learning process, tailored to personal learning needs and matching learning to practice.

Several factors need to be considered when determining the need for regulation of CPD:

- **Content:** What is new and required within the specific health system?
- **Relevance:** Does it meet societal expectations? Does it align with inter-professional and multidisciplinary working skills? Maintaining a collaborative relationship with industry without jeopardising unbiased continuing education.
- **Availability and access:** Who are the professionals that must access the materials and how best to engage with them – online, classroom, handbooks?
- **Recognition of learning:** Certification/recertification/formal/informal.

## CPD Stakeholders

Healthcare professionals, societies and colleges, regulators, educators, policy makers and healthcare authorities, patients and public are all CPD stakeholders. Meaningful coordination amongst them is fundamental to create an integrated and effective CPD system that meets the needs of the profession, patients and public. Open communication facilitates the identification of gaps in healthcare delivery, hence guiding societies to tailor CPD activities accordingly.

## Practically – how does it work?

Societies and Colleges as medical professional bodies were identified as having the main responsibility to set-up the CPD planning committee. This group should ideally include administrators, healthcare professionals, researchers, educationalists and experts in content. There should be individuals with healthcare expertise who are competent in leadership, collaboration, professionalism, communication, scholarship and advocacy.

They must be able to represent the members, ensure that educational content and instructional design match practical needs and make the CPD system more effective and accepted overall.

**Figure 1:**
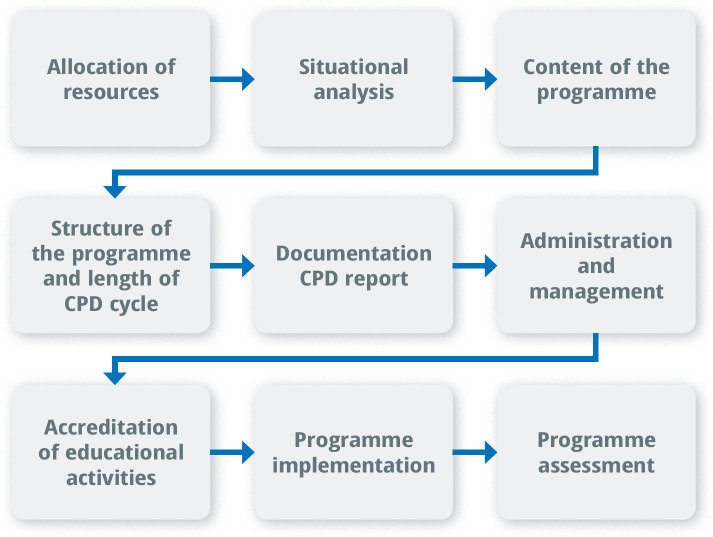
Nine-step plan to set up a CPD programme

The CPD committee are required to manage a range of tasks that include those shown on Figure 1 over the page.

## Setting and maintaining standards

Despite the worldwide diversity of approaching CPD, there is mutual agreement around a set of desirable and implementable global standards. This should stimulate the creation of CPD systems with a minimum requirement that are relevant to each region.

Considering the CPD integrated and holistic view, the International Council of Ophthalmology (ICO) created the “ICO Guide for Effective CPD/CME” a practical guide to the relevant core concepts and basic questions of CPD/CME bearing in mind the perspectives of participants, educators, regulators and providers^1^.

An effective CPD system (using the acronym SCAR) should encompass professional development as:

- **Systematic** by following a personal development plan based on the cyclic learning process involving reflection, planning, learning and assessing.
- **Comprehensive** by embracing competencies beyond medical expertise such as collaboration, scholarship, advocacy, leadership, communication and professionalism skills.
- **Accredited** structure through multifaceted learning interventions conducted in accordance with adult learning principles aiming for a change in practice.
- **Regulated** through demonstration of personal CPD progression.

**Figure:**
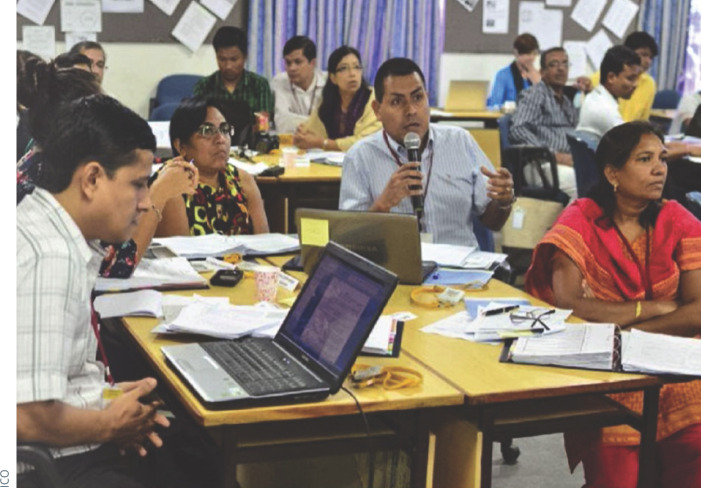
2015 Eyexcel Course in Madurai. INDIA

## Structuring CPD – a nine-step process (Figure 1)

The ICO has proposed a comprehensive approach to design a structured CPD system.

**Figure 2:**
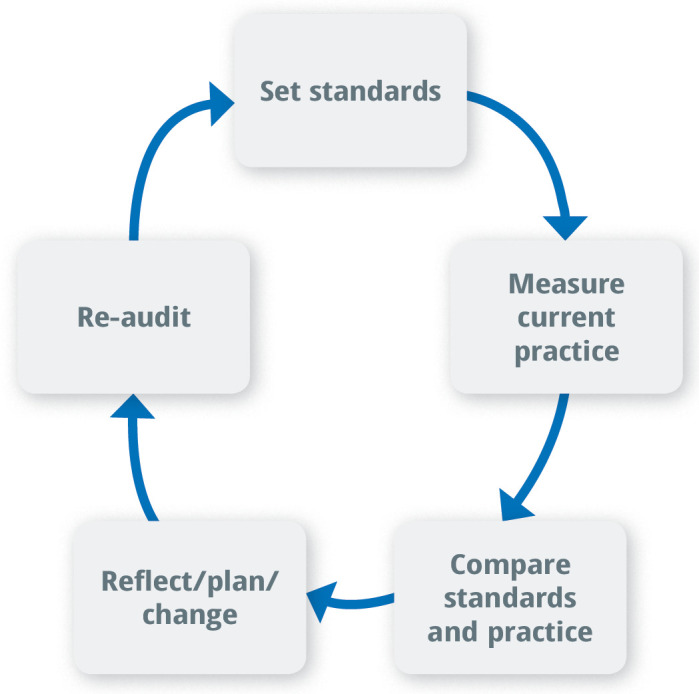
Clinical audits are effective assessment and learning tools comprising five cyclic steps

- Allocation of resources optimises the assignment of personnel and assets to meet requirements.
- Situational analysis allows understanding of current practices and the identification of gaps.
- Content of the programme should:
  - be **relevant**, based on a needs assessment and continuously updated to meet the public needs.
  - **flexible** to facilitate self-directed and practice based learning, allowing learning outside medical expertise, consider social or web-based or informal learning, and include inter-professional education.
  - be **delivered** through appropriate multimodal formats, adapted to personal learning styles and supported by faculty development to use the best educational strategies.
- The structure of the programme should take into account the length of the CPD cycle and its relationship to appraisal and licensure.
- Facilitate translation of new learning into practice by providing opportunities to disseminate and reinforce it. Documentation to demonstrate individual progression is an essential component of an effective CPD system.
- Administration and management should ensure a clear communication to the members about the programme structure, educational activities and important dates eventually using the society or college website as a cost-effective means. Individual notifications will help to ensure cycle completion, award the completion certificate or propose a remediation plan.
- Accredited educational activities should aim for a change in practice and the committee should provide accreditation guidelines. Commercial sponsorship should be regulated and biased education avoided (Figure 2).
- Programme implementation should emphasise that the best healthcare outcomes are tied to the concept that CPD is a professional requirement accomplished through lifelong learning and not an outward imposition.
- Programme assessment should include three components:
  1. system monitoring and self-regulation
  2. educational activities assessment
  3. professional regulation.

## Recording and assessing CPD

A robust CPD framework should include an assessment component. The system should ensure continuous monitoring and self-regulation.

All outcome levels of educational activities should be assessed: participation, satisfaction, learning (*knows, knows how, shows how*) performance (*does*) patient and community health. Educational activities should align: gap analysis – needs assessment – learning objectives – content/delivery format – assessment. Professional regulation resides in a clear demonstration of life long learning translated into practice. Either hard copy or online, portfolios are excellent assessment and learning tools, that emphasise the reflective component of an effective CPD system. They should mirror the activities undertaken, why they were pursued and how they helped to enhance practice. Societies and colleges should create and provide an effective system based on the portfolio to record the ongoing personal development plan.

Regulation should be informed by the profession and viewed as the means to effectively demonstrate professional progression and accountability. CPD systems are moving towards the demonstration of a mandatory set of requirements, either based on credit units awarded by the time spent on educational activities or on peer review based on documentation. In either one the inherent professional obligation to society should remain as a primary imperative.

## Conclusion

CPD should be a local effort, depending on local disease patterns and health system requirements. Of all medical education stages, CPD is the least formally structured and can be the most complex to create and assess given the diversity of curricula, educators, regional healthcare needs, professional aspirations, complexity of working environment and multiple stakeholders.

CPD is a primary obligation to the patients and the public. The paradigm has been shifting to place the healthcare team in the centre of CPD good practice. Professional bodies should maintain and reinforce their core role in providing CPD guidelines, standards, and benchmarks creating systems to effectively record and share information and documents among members. Professional bodies should involve all stakeholders engaged in meeting the public healthcare needs and build a CPD system that is efficient, transparent, credible, accountable, affordable, easy to manage and administer, driven within the profession, and flexible to the community health care needs. The educational programme should be exempt from industry influence and cover multimodal practice-based activities aiming for practice change.

Managing CPD systems should ideally include the audit and portfolio as components and be regularly self-assessed for improvement. To build this holistic view of CPD, the CPD planning committee members should themselves develop competencies of leadership, professionalism, communication skills, advocacy, scholarship, and collaboration.

**Reasons for keeping up to date**

Some of these scenarios may strike a chord with readers. Undertaking CPD to fill in the knowledge gaps may ensure a happier experience both for the patient and eye care practitioner.

**Clinical knowledge**

- Do you lack confidence when faced with patients with certain conditions?
- Can you distinguish between optic atrophy and advanced glaucoma?
- Have you ever missed new vessels when examining the fundus of a patient with diabetes?
- Do you know what to do when faced with a patient who has just gone blind?
- Can you competently carry out a visual field assessment before ordering a CT or MRI scan?
- Can you interpret a B-scan?
- Can you read an OCT?
- Can you calibrate an applanation tonometer?
- Have you ever administered the wrong dose of a drug to a patient?
- Are you up to date with drug interactions?
- If you witness a new procedure, do you write down/draw what you have seen?
- Are you able to resuscitate a patient who has collapsed in the clinic?

**Figure:**
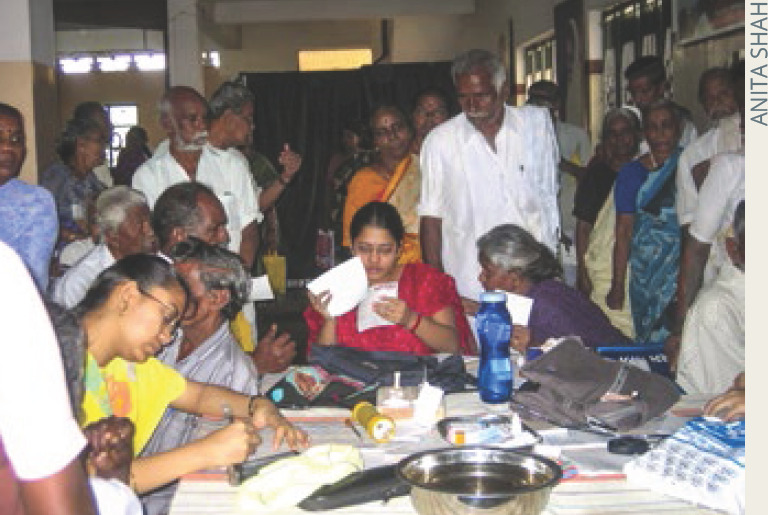
Time management is important when the clinic is very busy. INDIA

**Procedural skills**

- Do you feel under-prepared when starting a particular surgical procedure?
- Are you familiar with all the settings on all the equipment that you use?
- Do you know what to do if your patient loses vitreous during cataract surgery?
- Do you know how to make up the correct concentrations of intravitreal antibiotics?
- Have you ever pitted the IOL when doing a YAG laser?
- Can you tell a patient your cataract surgical outcomes?

**Personal development**

- Do you want to be an effective leader?
- Do you want to be able to effect change?
- Do you aspire to be as good as one of your colleagues?
- Are you willing to learn from others in the team?
- Do you want to be on time for your clinic?

**If things go wrong**

- If things have gone wrong – do you know who to talk to?
- Do you discuss ‘failures’ or disappointments with your colleagues?
- Do you know how to deal with an official complaint concerning your management?
- Do you know how to report a ‘clinical incident’ or ‘near miss’?

*Nick Astbury, Clinical Senior Lecturer, ICEH*
